# Role of Millets for Food Security Under Climate Change

**DOI:** 10.1002/pei3.70128

**Published:** 2026-02-20

**Authors:** Bibas B.K., Sneha Dahal, Manisha Koirala, Rashmi Poudel, Bishnu Prasad Kandel

**Affiliations:** ^1^ Institute of Agriculture and Animal Science Tribhuvan University, Lamjung Campus Kathmandu Nepal

**Keywords:** climate resilience, food security, millets, small‐grain cereals, sustainable global food

## Abstract

The accelerating impacts of climate change pose significant threats to global food security, highlighting critical vulnerabilities within the agricultural system. As greenhouse gas emissions continue to rise, global temperatures have increased by 0.6°C over the 20th century, with projections indicating further increases of 0.1°C–2°C per decade. These trends are expected to reduce crop productivity and food availability, potentially leaving up to three billion people undernourished by 2050. Therefore, diversification of agricultural cropping systems is crucial, especially through the incorporation of underutilized and resilient crops like millets. Millets, a group of small‐seeded grasses, exhibit tolerance to both biotic and abiotic stress and can thrive under harsh environmental conditions such as poor soil fertility, low rainfall, drought, and salinity, making them particularly suitable for climate‐vulnerable agro‐ecosystems. As C4 crops, they have high photosynthetic efficiency and shorter growth durations than many C3 staples. These small‐grain cereals are rich sources of gluten‐free proteins, dietary fiber, vitamins, and essential minerals, and can contribute to improved nutritional security. Additionally, bioactive compounds present in grains offer therapeutic properties against various disorders and diseases, highlighting their promising nutraceutical potential. Furthermore, advances in biotechnological approaches, including molecular markers and genetic improvement techniques, offer opportunities to enhance stress tolerance and nutritional traits. This review provides insights into millets' role in food security, nutrition, and pharmaceuticals, examines their stress‐adaptive traits, and discusses advances in genomics and biotechnology. Although it integrates findings from previous studies, this review presents a new integrative perspective focused on enhancing millet cultivation within agricultural systems.

## 1 Introduction

Global climate change has emerged as one of the most critical challenges to global food security, exerting far‐reaching impacts on agricultural systems worldwide. These impacts are manifested through rising temperatures, reductions in snow and ice, and an increased frequency of heavy precipitation in many regions (IPCC 2014). Such changes also disrupt hydrological cycles, intensifying droughts by increasing their frequency, duration, and severity worldwide (Mukherjee et al. 2018). In this context, millets are particularly valuable, as they possess inherent tolerance to drought and high temperatures, which is attributed to their unique morphophysiological, molecular, and biochemical traits, enabling them to withstand environmental stresses more effectively than major cereal crops (Yadav et al. 2012; Patan et al. 2024). Along with anatomical and physiological adaptations, dehydration responses enable millets to tolerate very low critical leaf water potentials (−1.22 MPa) (Tiwari et al. 2022). As C4 plants, millets exhibit high photosynthetic efficiency, recognized as crops with low carbon and water footprints (Yadav et al. 2024), and strong adaptability to diverse ecological conditions, which enables them to thrive across arid, semiarid, subtropical, and tropical climates (Khanal 2023). Additionally, they can be cultivated across a diverse altitude range, from 60 to 3500 m (Joshi 2017), and are capable of flourishing even on marginal lands, where other crops struggle (Joshi et al. 2023). This resilience and adaptability have contributed to their widespread cultivation globally. Currently, millet cultivation occurs in 93 countries, with just seven nations accounting for over 1 million hectares of farmland, and developing countries contributing more than 97% of global millet production and consumption (Meena et al. 2021). Their extensive cultivation and consumption underscore their importance as a staple crop, particularly in developing regions.

**FIGURE 1:**
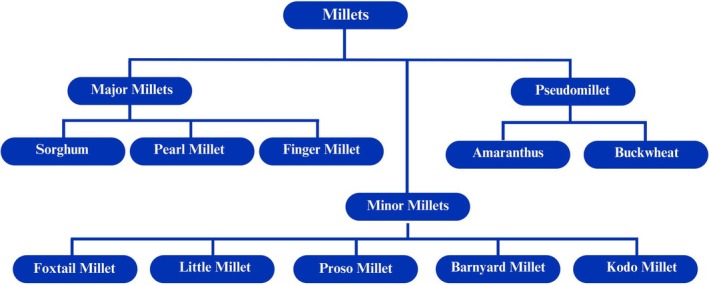
Classification of millets. Adapted with permission from Chandra et al. (2022).

Millets are a group of small‐grain cereals belonging to several grass species (FAO 2023) and are classified into major millets, minor millets, and pseudomillets (Figure 1). Major millets comprise sorghum ( *Sorghum bicolor* ), pearl millet ( *Pennisetum glaucum* ), and finger millet ( *Eleusine coracana* ), whereas minor millets consist of several cultivated species, including little millet ( *Panicum sumatrense* ), proso millet ( *Panicum miliaceum* ), barnyard millet ( *Echinochloa esculenta* ), kodo millet ( *Paspalum scrobiculatum* ), browntop millet (*Urochloa ramose*), and foxtail millet ( *Setaria italica* ) (Rawat et al. 2021). Similarly, buckwheat (*Fagopyrum esculentum*) and amaranthus ( *Amaranthus cruentus* ) are classified as pseudomillets. These grains are rich in essential micronutrients, including calcium, iron, dietary fiber, antioxidants, and amino acids, and have a low glycemic index, making them vital for the nutrition security of the poor (Mal et al. 2010; Saleh et al. 2013; Kam et al. 2016). Their exceptional nutrient profile (Anbukkani et al. 2017), along with health benefits and resilience to harsh climates, has established them as “smart food” crops for the future (Joshi et al. 2023). In addition to their resilience, millets have excellent storage qualities, retaining their viability for extended periods without susceptibility to common pests, ensuring food security and stable reserves.

Despite their clear promise, millets have been largely overlooked in recent decades, with few agronomists, economists, or policymakers giving them the attention they deserve. As millets lose priority in farmers' fields, much of the ancient knowledge associated with their cultivation has been lost (FAO 2023). Between 1961 and 2018, the global area dedicated to millet cultivation decreased by approximately 25.71% (Meena et al. 2021). This decline suggests a possible influence of irrigation, urbanization, and globalization. The expansion of irrigation systems favored crops such as rice and wheat, which respond better to government support and more stable returns (Timilsina et al. 2025). Urbanization and changing food markets further reduced demand for millet‐based foods, leading to the gradual displacement of millets from production systems and culinary practices (Dhakal et al. 2023). As global attention increasingly focuses on climate change and its impact on farming and food production, it is crucial to explore the genetic diversity of millet landraces and deepen our understanding of their drought‐resistance traits and adaptive mechanisms (Poudel 2023). Recognizing the nutritive and health benefits of millets and their potential for production under harsh and changing climatic conditions (Mal et al. 2010; Rawat et al. 2021), the United Nations General Assembly declared 2023 as the International Year of Millets (IYM). This initiative aligns strongly with the United Nations 2030 Agenda for Sustainable Development and directly contributes to six of the 17 Sustainable Development Goals (SDGs): Zero Hunger (SDG 2), Good Health and Well‐being (SDG 3), Decent Work and Economic Growth (SDG 8), Responsible Consumption and Production (SDG 12), Climate Action (SDG 13), and Life on Land (SDG 15). By mainstreaming millets into policy frameworks and raising awareness about their cultivation and utilization, IYM supports efforts to achieve these SDGs while promoting sustainable agriculture and food security (FAO 2023).

This study aims to explore the multifaceted role of millets in addressing food security and their adaptive responses to climate change‐induced stresses. It highlights the contributions of millets to global food systems, emphasizing their nutritional value and pharmaceutical potential. It further examines the adaptive traits that enable millets to withstand biotic and abiotic stresses, such as drought, poor soil quality, pest infestations, and disease attacks, which are increasingly exacerbated by climate change. The review also delves into advancements in millet genomics and biotechnology and provides various strategies to enhance their production and integrate them into mainstream agricultural practices.

## 2 Climate Change and Millet Production

Over the past five decades, global agricultural production has generally increased. However, this growth is proving unsustainable, primarily due to the adverse impacts of climate change (IPCC 2022). Drought, desertification, rising temperatures, and the increasing frequency of extreme climatic events are key factors hindering efforts to achieve the Sustainable Development Goals (IPCC 2014). This poses a significant threat to sustainable agriculture by disrupting crop development, hindering farming operations, and diminishing overall yields. For instance, wheat production is expected to decrease by 3%–10% with every 1°C increase in temperature (You et al. 2009). Similarly, Peng et al. (2004) reported adverse effects of high night temperatures (HNT) on rice production during the growing season, with yields declining by 10% for every 1°C increase in HNT. Moreover, these climate‐related challenges are compounded by the agriculture sector, a significant contributor to global greenhouse gas emissions. Among staple crops, wheat, maize, and rice have high Global Warming Potentials, emitting 3968, 3427, and 3401 kg CO_2_ eq. ha^−1^, respectively. In comparison, millets have substantially lower Global Warming Potentials, with pearl millet emitting 3218 kg CO_2_ eq. ha^−1^ and sorghum emitting 3358 kg CO_2_ eq. ha^−1^ (Jain et al. 2016; Wang et al. 2018). Similarly, carbon equivalent emissions are highest for wheat (1042 kg C ha^−1^) and lowest for millet (878 kg C ha^−1^) (Jain et al. 2016). These findings highlight that millet is a promising option for reducing the agriculture sector's contribution to global warming.

**FIGURE 2:**
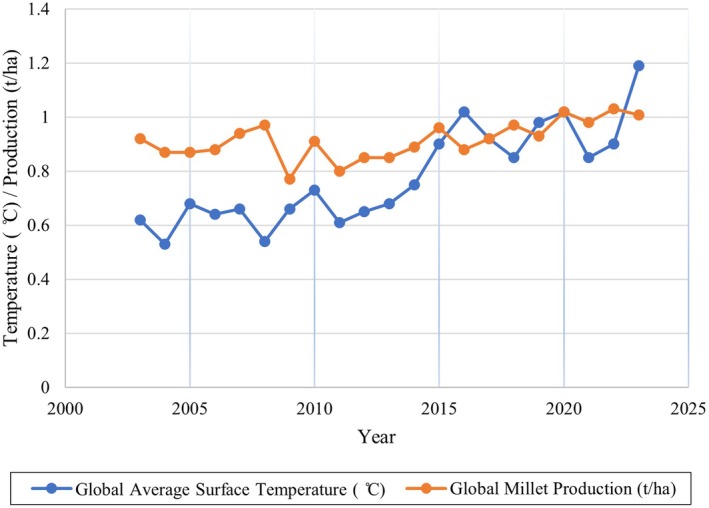
Global average surface temperature and global millet production (FAOSTAT 2024; NASA 2024).

As shown in Figure 2, Earth's average surface temperature has been rising since 2011. According to NASA 2024 was the hottest year on record (1.28°C), and the past decade has been the warmest in recorded history. Similarly, the global trend of millet production has been increasing alongside the rise in Earth's surface temperature (Figure 2), likely due to millet's well‐developed deep root systems and drought tolerance capacity. Improvements in irrigation practices, cultivar development, fertilizer use, agronomic management, value‐added products, and supportive policy incentives also play important roles in enhancing millet productivity (Yadav et al. 2024). Some studies have indicated that rising temperatures up to certain thresholds in dry regions can enhance millet production. For instance, Cao et al. (2010) reported that millet yields increased from 30 to 121 kg ha^−1^ with temperature rises across various cities in China. Similarly, the CSM‐CERES‐Pearl Millet simulation model examined by Singh et al. (2017) demonstrated that millet yields improved by 6% after drought (lower limit of soil water availability) and 8% after heat simulation (increased from 27°C to 29°C). However, exposure to temperatures exceeding the threshold leads to significant reductions in grain yield and physiological functions due to heat‐induced stress and cellular damage (Opole et al. 2018). Djanaguiraman et al. (2018) found that reproductive stages in pearl millet are particularly sensitive to high‐temperature stress (≥ 36°C/26°C), resulting in reduced seed number, grain weight, and yield due to increased reactive oxygen species and decreased antioxidant activity. However, pearl millet and sorghum have been reported to tolerate high temperatures of up to 42°C during the flowering stage (Gupta et al. 2015). Finger millet also exhibited significant yield and physiological declines when exposed to temperatures above 35°C during reproductive stages, with reduced chlorophyll content and photosystem II activity, highlighting critical sensitivity at booting, panicle emergence, and flowering (Opole et al. 2018).

## 3 Role of Millets for Food Security

**FIGURE 3:**
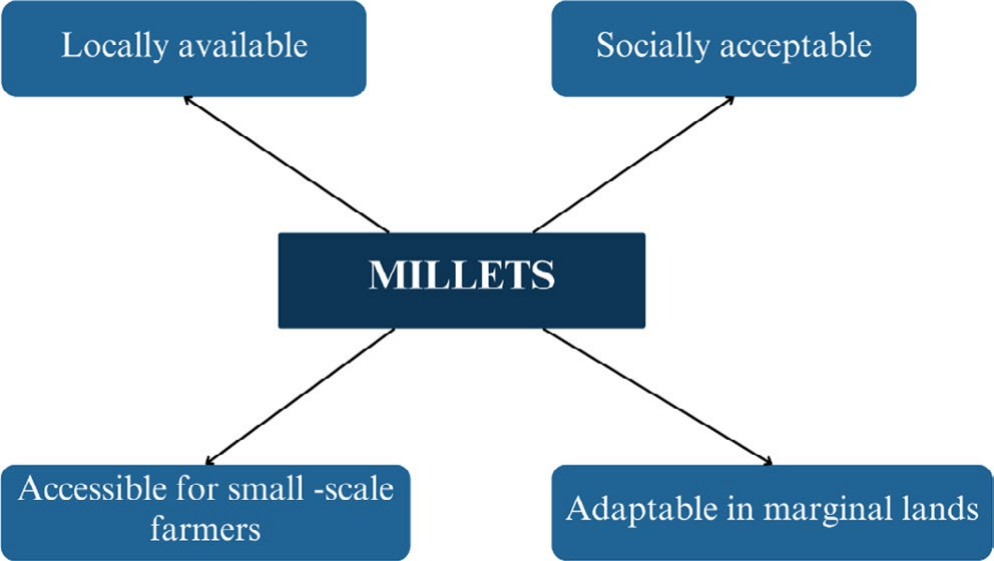
Role of millets for food security.

Millets are increasingly recognized as a crucial component in enhancing food security, especially in regions facing agricultural challenges due to climate change and economic instability. These crops possess valuable attributes: they are locally available, socially acceptable, accessible to small‐scale farmers, and highly adaptable to marginal areas, making them pivotal for ensuring food security. Figure 3 illustrates the role of millets in ensuring food security, which is further discussed below:

- Availability: Millet species exhibit a wide range of maturity durations depending on the species and variety. For instance, proso millet matures within 60–100 days (Habiyaremye et al. 2017), while finger millet varieties mature between 90 and 120 days (Singh et al. 2017). This variability allows farmers to stagger planting and harvesting cycles to ensure year‐round availability and better align production with local climatic conditions.
- Acceptability: Millets have been utilized in diverse culinary applications across various cultures. Archaeobotanical studies suggest that these grains were used as a food source as early as the Iron Age in southern India (Bates et al. 2021). Today, they are commonly prepared as flatbreads, porridges, rotis, dumplings, and beverages. Certain millets, such as pearl millet, sorghum, and finger millet, are traditionally cooked and fermented with curd and water to enhance nutrient absorption (Sheethal et al. 2022).
- Affordability: Millet farming is affordable and ideal for small‐scale farmers due to low input needs and adaptability (Hulse et al. 1980). It also offers higher income from both grain and straw compared to major cereals, which depend heavily on external inputs (Muthamilarasan and Prasad 2021). Millet costs about 40% less than maize, making it an economical and gluten‐free cereal option (Kumari et al. 2024). Due to its resistance to drought, pests, and diseases (Purohit and Palai 2020), along with minimal irrigation needs (Mude et al. 2020), it may help farmers achieve better economic returns even with limited agricultural inputs.
- Adaptability: Millets are well‐suited for cultivation in marginal lands and water‐scarce regions (Gairhe et al. 2021). Furthermore, small millets are often intercropped with legumes to improve soil health, as intercropping boosts enzymatic activity, air circulation, and water retention (Ananthi et al. 2024). Incorporating such resilient crops not only enhances agricultural productivity but also supports ecosystem protection (Kumar et al. 2022). Due to their remarkable adaptability, millets are often referred to as “Future Smart Crops” or “Crops for Risk Diversification” (Gairhe et al. 2021).

### 3.1 Millets for Nutritional Security

The concurrent rise in the global population and decline in crop diversity underscores the urgent need for nutrient‐dense crops like millets. By 2050, the world population is projected to reach 9 billion, with approximately 3 billion people at risk of starvation and malnourishment due to declining crop production rates (Kumar et al. 2022). Alarmingly, only 20% of crops among 7000 edible plant species contribute to 90% of the global food supply (Muthamilarasan and Prasad 2021). Moreover, three staple crops: rice, wheat, and maize, account for 60% of global cultivation, leaving nutrient‐rich crops like millet underutilized. Nevertheless, the significant potential of millets to ensure nutritional security has gained recognition in recent years.

Millets currently occupy a considerable share of global production volume and area, though they remain subordinate to major cereals worldwide. The International Crops Research Institute for the Semi‐Arid Tropics (ICRISAT) ranks finger millet as the sixth most important crop in terms of production (Kumar et al. 2018). In countries like India, crops such as sorghum, pearl millet (bajra), and finger millet occupy less production area compared to maize, rice, and wheat (Devi et al. 2014). In Africa and Asia, over 90 million people rely exclusively on millet to meet their nutritional needs (Srivastava and Reddy 2023). Despite growing recognition of their value, millets are still often labeled as “poor man's crops” or “ancestors' crops.”

Millets play a crucial role in combating hunger and promoting good health due to their rich nutritional profile, which includes protein, crude fiber, B‐complex vitamins, vitamin E, and essential minerals such as calcium, magnesium, iron, potassium, and phosphorus (Tables 1 and 2). The resistant starch in small millets aids in breaking down complex carbohydrates into simpler forms, providing 2000–3000 cal per person per day on average (Muthamilarasan and Prasad 2021). Additionally, the high dietary fiber and non‐starch polysaccharide content in millets contributes to maintaining a healthy body weight (Nikale and Millet 2023). Regular inclusion of small millets in diets has been linked to significant reductions in malnutrition, stunting, and wasting among children and adolescents (Srivastava and Reddy 2023).

**TABLE 1:** Nutritional composition of millets (per 100 g).

Nutrients per 100 g						
Millets	Energy (Kcal)	Carbohydrates (g)	Protein (g)	Fats (g)	Crude fiber (g)	Minerals (g)
Finger millet	328	72.0	7.3	1.3	3.6	2.7
Pearl millet	361	67.5	11.6	5.0	1.2	2.3
Foxtail millet	331	60.9	12.3	4.3	8.0	3.3
Proso millet	341	70.4	12.5	1.1	2.2	1.9
Kodo millet	309	65.9	8.3	1.4	9.0	2.6
Little millet	341	67.0	7.7	4.7	7.6	1.5
Sorghum	349	72.6	10.4	1.9	1.6	1.6
Barnyard millet	307	65.5	6.2	2.2	9.8	4.4

*Source:* Gopalan et al. (2016).

**TABLE 2:** Mineral composition of millet (per 100 g).

Nutrients per 100 g										
Millets	Fe (mg)	Ca (mg)	Zn (mg)	P (mg)	K (mg)	Cu (mg)	Cl (mg)	Mg (mg)	S (mg)	Na (mg)
Finger millet	3.9	344	2.3	283	408	0.47	44	137	160	11.0
Pearl millet	8.0	42	3.1	296	307	1.06	39	137	147	10.9
Foxtail millet	2.8	31	2.4	290	250	1.40	37	81	171	4.6
Proso millet	0.8	14	1.4	206	113	1.60	19	153	157	8.2
Kodo millet	0.5	27	0.7	188	144	1.60	11	147	136	4.6
Little millet	9.3	17	3.7	220	129	1.00	13	133	149	8.1
Sorghum	4.1	25	1.6	222	131	0.46	44	171	54	7.3
Barnyard millet	5.0	20	3.0	280	—	0.60	—	82	—	—

*Source:* Gopalan et al. (2016).

Millets are rich in amino acids and fatty acids that are often deficient in starch‐rich crops. For example, the essential amino acid content of finger millet is 44.7%, surpassing the FAO reference value of 33.9% (Kumar et al. 2018). It also contains higher levels of amino acids such as phenylalanine, isoleucine, and leucine, which are limited in other starch‐rich cereals (Nikale and Millet 2023). Small millets like fonio millet ( *Digitaria exilis* ), commonly cultivated in Africa, are abundant in methionine and cysteine. Similarly, teff millet holds significant nutritional value due to its higher lysine content—an amino acid deficient in most major cereals (Pramitha et al. 2023). Millets also provide essential fatty acids in both free and bound forms, including palmitic, linoleic, and oleic acids in free form, and phosphatidyl serine, monogalactosyl diglyceride, and phosphatidyl choline in bound form (Sarita 2016). Consequently, millets are an excellent source of macronutrients such as carbohydrates, protein, fats, as well as their simpler forms. Besides, they are an excellent dietary source due to their abundance of micronutrients, such as vitamins and minerals. They are particularly rich in folic acid, which can help alleviate anemic disorders (Tiwari et al. 2023). Millets contain nearly twice the calcium found in rice, with finger millet offering up to 30 times the calcium content of rice (Rotela et al. 2021). Additionally, yellow pearl millets are abundant in essential minerals like zinc and copper, as well as vitamin A (retinol) (Rotela et al. 2021). Enriched with both macro‐ and micronutrients essential for growth and development, incorporating millets into diets can play a significant role in reducing malnutrition (Gahalawat et al. 2024). However, their nutraceutical value has been recognized in recent years, leading to the designation of millets as “Nutri‐cereals” (Singh et al. 2024) or “Miracle Grains” (Bordoloi et al. 2023).

### 3.2 Millets for Health Benefits

A reciprocal relationship exists whereby the regular consumption of polyphenol‐rich millets is associated with potential health‐promoting effects (Rudrapal et al. 2022; Zekrumah et al. 2023); however, the extent of these clinical outcomes requires further validation. As an alkaline food, millets support the function of digestive enzymes and help maintain optimal body pH (Sarita 2016). For instance, foxtail millet exhibits antiulcer properties by reducing plasma and mucosal TBARS (Thiobarbituric acid reactive substances) levels and boosting gastric nonprotein sulfhydryl (NPSH) digestive enzyme activities (Shi et al. 2017). Insoluble fibers from millets help eliminate toxins from the digestive system, increase fecal volume, and improve intestinal movement by boosting water retention (Weisburger et al. 1993; Basen and Kurrer 2021). Similarly, soluble fibers such as beta‐glucans, arabinoxylans, and pectins form gels by absorbing water, increasing food viscosity, slowing nutrient absorption, and helping to reduce dyslipidemia and postprandial glucose levels (Jacob et al. 2024). Millets release sugars slowly and thus have a low glycemic index (GI), with values ranging from 54 to 68 for millets such as foxtail, little, finger, and pearl millet (Patil et al. 2015), which is associated with a reduced postprandial glucose response; therefore, they are considered an ideal food for patients with gastrointestinal diseases and non‐insulin‐dependent diabetes mellitus (Anju and Sarita 2010; Kumar et al. 2022). Similarly, Inhibitors like aldose reductase reduce sorbitol accumulation, helping to prevent diabetes‐induced cataracts (Sarita 2016). Phytochemicals, especially quercetin present in the polyphenolic compounds of finger millet seed coats, have shown noncompetitive inhibition and are considered potential natural substitutes for synthetic aldose reductase inhibitors (Lee and Kim 2001; Kim et al. 2002; Chethan et al. 2008). The phenolic antioxidants in millet extracts protect insulin‐producing cells from damage and promote wound healing (Devi et al. 2014). Millets also support the immune system, as they are rich in antioxidants, and help combat chronic diseases such as cardiovascular disorders. Antioxidants like β‐glucan and γ‐aminobutyric acid (GABA) in barnyard millet contribute to reduced blood lipids (Muthamilarasan and Prasad 2021). Daily intake of finger millet increases the activity of antioxidant enzymes, such as catalase and glutathione peroxidase, and helps slow the aging process (Rotela et al. 2021). Furthermore, finger millet consumption reduces skin stiffness, tendon and ligament rigidity, and collagen cross‐linking (Saleem et al. 2023).

Table 3 represents several health benefits associated with millet consumption. Millets contain bioactive compounds such as phytates, tannins, and phenolic acids, which have been shown to reduce the risk of breast and colon cancer (Dey et al. 2022). Quercetin, a specific polyphenol extract from finger millet, has been identified as a potential alternative to artificial androgen receptor inhibitors (Chethan et al. 2008) and has demonstrated effective inhibitory activity against aldolase (Shobana et al. 2009). Proso millet and sorghum extracts, which contain ferulic acid and chlorogenic acid, are known to inhibit the proliferation of cancer cells, making them promising anticancer supplements (Shobana et al. 2009; Zhang, Liu, and Niu 2014). Millet consumption also benefits the nervous system and sleep regulation (Premachandran et al. 2023). Due to its tryptophan content, a precursor of neurotransmitters like serotonin, millet helps regulate the sleep cycle (Jamal et al. 2023). Lecithin, found in higher concentrations in Kodo millet, strengthens the neurological system (Nikale and Millet 2023). Additionally, the abundance of magnesium in millets reduces the risks of heart attacks and migraines (Sarita 2016; Ambati and Sucharitha 2019). As a result, the use of millets in reducing disease risk is gaining attention due to their significant pharmacological and phytochemical potential.

**TABLE 3:** Health benefits of millet species.

Millet species	Chemical components	Functional role	References
Pearl millet	Antioxidants, flavonoids	Reduces oxidative stress and inflammation, and neurodegenerative diseases (NDs)	Rathore et al. (2017)
Finger millet	Methanolic extract	Prevents glycation and crosslinking of collagen, reduces complications of diabetes and aging due to the presence of free radical scavengers	Hegde et al. (2002)
Foxtail millet	Protein concentrate	Higher adinopectin that reduces proinflammatory responses in cardiovascular tissues	Choi et al. (2005)
Proso millet	Phenolic extracts	Lipid peroxidation inhibition, elevated cholesterol metabolism reducing serum triglycerides	Chandrasekara and Shahidi (2011)
Kodo millet	Antioxidants	Free radical quenching potential	Hegde and Chandra (2005)
Barnyard millet	Fatty acids	Decrease cholesterol and phosphorus levels, improve metabolic efficiency, and energy conversion	Veena et al. (2005)
Sorghum	Tannins, polyphenols	Anticarcinogenic and antimutagenic	Grimmer et al. (1992)
Foxtail millet	Phenolics and carotenoids	Prevents cardiovascular and geriatric disease, inhibits the growth of human breast and liver cancer cells.	Zhang and Liu (2015)
Finger millet	Polyphenols	Prevents the accumulation of sorbitol, reduces the risk of diabetes induced cataract diseases	Chethan et al. (2008)
Little millet	Kaempferol	Lowers the risk of chronic diseases	Pradeep and Sreerama (2018)
Kodo millet	Campesterol	Reduces cholesterol, shows anticarcinogenic and chemopreventive effect	Choi et al. (2007)

### 3.3 Value Addition and Product Diversification of Millets

As climate change intensifies pressures on agricultural productivity and dietary diversity, millets have emerged as a significant potential for value addition by incorporating them into a diverse range of traditional and modern food products. Their superior nutritional profile has drawn the attention of research institutions worldwide, leading to increased efforts to expand their utilization in processed food products (Kumar et al. 2018). Traditionally, millets have been used to make foods such as *ragi mudde* (a soft dough ball eaten with curry) and *ragi dosa* (a type of pancake), which are still common in India (Hema et al. 2022). Due to their nutritional benefits, taste, and safety, they are commonly used as weaning foods for babies (Chakraborty and Chakraborty 2021). Traditional fermented beverages derived from millets are popular in various countries. For instance, *Tongba*, which is made from finger millet, is widely consumed in the Himalayan region of Nepal and possesses therapeutic properties that help alleviate various high‐altitude illnesses (Majumder et al. 2022; Karki et al. 2023). *Chyang*, prepared from Kodo millet, is a widely consumed mild‐alcoholic beverage in the Sikkim and Darjeeling hills of India (Ankita and Seth 2025). *Bushera*, a fermented beverage made from sorghum and millet, is widely consumed by young children and adults in Uganda as a nutritious, low‐cost meal replacement and an excellent source of energy (Hawaz et al. 2025). Similarly, *Borde*, a traditional fermented low‐alcoholic beverage made from finger millet and sorghum, is common in the southern and western parts of Ethiopia (Fentie et al. 2020). Mothers are encouraged to drink large amounts of *Borde* after giving birth, as it is traditionally believed to improve breastfeeding (Debebe et al. 2017). In Tanzania, *cipumu*, prepared from finger millet, holds cultural significance in rituals and provides an important source of nutrition for the local community (Badi et al. 1990).

Fermented foods like *dosa and idli*, commonly consumed in South India, serve as excellent breakfast options, offering an alternative to rice in their preparation (Chakraborty and Chakraborty 2021). *Koozh*, prepared from finger millet and pearl millet, is renowned for its flavor and nutritional value, and is commonly consumed as a breakfast meal in the Tamil Nadu state of India (Thirumangaimannan and Gurumurthy 2013). Similarly, several non‐fermented millet‐based products are also common in many countries. *Dhindo*, a thick, pasty Nepalese porridge prepared from finger millet, serves as an excellent food option to a large number of diabetic patients (Sharma, Pudasaini, et al. 2020). *Kisra*, a flatbread prepared from sorghum or millet, is a staple food of Sudan (Badi et al. 1990). When made with 25% chickpeas, it provides an adequate level of protein for children over the age of 9 months (FAO/WHO 1985). Similarly, *Roti* (flatbread) and *gatka* (porridge) made from Jowar flour serve a crucial role for the livelihood and food security of indigenous groups in India (Ravula et al. 2022).

The growing popularity of millet‐based ready‐to‐eat foods offers fiber‐rich and low‐glycemic index (Porwal et al. 2023) options for health‐conscious urban populations (Arya and Bisht 2022). Millets are an excellent ingredient for formulating diabetic‐friendly foods due to their high content of resistant starch, soluble and insoluble fiber, minerals, vitamins, and antioxidants (Amadou et al. 2013). Multigrain flour, made from a combination of wheat and finger millet at a ratio of 3:7, yields favorable semifinished products suitable for making *chapattis* (Chakraborty and Chakraborty 2021). It has been shown to enhance flavor and exhibit antidiabetic properties, making it beneficial for individuals with diabetes (Kang et al. 2008). Traditional recipes such as *biryani, kheer*, and *khichdi* prepared with foxtail and barnyard millets demonstrated higher nutritional content and better sensory acceptability compared to their rice‐based versions (Verma et al. 2015). Millet grains are a gluten‐free source of protein; hence, they are not ideal for use as the sole ingredient in food product formulations and are better suited as a component in the preparation of bakery items (Chakraborty and Chakraborty 2021). Millet grains are milled into fine flours that can be used alone or blended with other flours for baking bread, biscuits, muffins, and cakes. In developed nations, a wide range of convenience foods, including extruded items, are widely consumed. These extruded products consist of spaghetti, macaroni, vermicelli, noodles, pasta, and similar items (Saraswathi and Hameed 2022). The Indian Institute of Millets Research (IIMR) has developed over 50 millet‐based value‐added technologies, emphasizing millet flour blends, bakery products, malted mixes, and extruded items to meet urban demand (Dayakar et al. 2016).

## 4 Contribution of Millets for Adaptation Against Climate Change

The heavy reliance on rain‐fed agriculture is facing increasing challenges due to the escalating frequency of droughts, reduced rainfall, and a rise in pests and diseases (Yvonne et al. 2016). As water sources dry up and rainfall patterns become more erratic due to climate change (Harrison et al. 2014), farmers are turning to millet species, such as sorghum, foxtail millet, and pearl millet, for cultivation in rainfed areas (Fischer et al. 2016). The global impact of climate change has led to a decline in the productivity of major staple crops, highlighting the need for millet crops in agricultural systems to promote climate‐smart practices. As C4 plants, millets are highly efficient in photosynthesis, have a shorter growing season, accumulate dry matter more efficiently, and exhibit excellent tolerance to heat and drought stress. Similarly, they have a low carbon footprint and are more environmentally friendly compared to other cereals (Gupta et al. 2025).

### 4.1 Millets Against Abiotic Stress

The adaptability of Pearl millet is defined by its capacity to endure abiotic stresses such as soil moisture stress, saline soils, low soil nutrient levels, and heat stress during seedling and flowering, as well as seed set stages (Rai et al. 2008; Sharma, Sharma, et al. 2020). It is often called the “camel” of crops because of its remarkable drought tolerance, and its seedlings are capable of producing heat shock proteins when exposed to heat stress (35°C–45°C) (Das and Rakshit 2016). Vapor Pressure Deficit (VPD), which serves as a useful indicator of evaporation demand as moisture transitions from the surface to the atmosphere, can also serve as a dynamic measure of drought conditions and be employed to detect transient drought episodes (Gamelin et al. 2022). Sorghum and pearl millet minimized water losses by reducing their leaf area as a response to drought stress when cultivated under elevated VPD (Choudhary et al. 2020). One of the characteristic features of abiotic stress resistance in pearl millet can be attributed to the presence of glutathione reductase (GR), a unique enzyme that performs a significant part in regulating the homeostatic redox balance at the cellular level since excess reactive oxygen species (ROS) are a common result of abiotic stress on plants (Achary et al. 2015). These findings suggest that inherent enzymatic mechanisms and physiological adjustments help millet species thrive better in the face of abiotic stress imposed by drought conditions.

Various research works have shed light on the potential of finger millet, standing out as a valuable crop from which tolerant alleles and genes associated with resilience to stress can be sourced (Ramakrishna et al. 2018). For instance, EcbZIP17, the membrane‐tethered transcription factor derived from finger millet, was found to play a crucial function by promoting crop growth and bolstering abiotic stress tolerance. Research findings suggest that under optimal conditions, EcbZIP17 enhances plant growth through brassinosteroid signaling. Furthermore, EcbZIP17 imparts tolerance to various environmental stresses by engaging the endoplasmic reticulum (ER) signaling pathways. The expression analysis revealed the presence of EcbZIP17 in overall vegetative parts of the plant, such as the leaf, shoot, root, panicle, and germinated seed. Interestingly, the expression of EcbZIP17 was upregulated under all stress treatments, with notable increases observed after 24 h of exposure to heat, dehydration, abscisic acid (ABA), and hydrogen peroxide (H_2_O_2_) stresses. This indicates the potential role of EcbZIP17 in facilitating plant adaptation and resilience under adverse environmental conditions (Ramakrishna et al. 2018). The findings of Xing et al. (2022) indicate that histone acetyltransferase in foxtail millet (SiHAT) has certain functions in responding to abiotic stress and viral infection. Histone acetylation may influence the growth and development of the plant and its stress reaction. Yugu1 genome analysis identified 24 HATs in foxtail millet (Xing et al. 2022). Similarly, a total of 25 ARF (Auxin Response Factors) genes have been identified in foxtail millet (Nadeem et al. 2020), which control embryogenesis, leaf growth, senescence, and root and fruit development by regulating auxin response genes (Wilmoth et al. 2005). Plant lipoxygenases (LOXs) are nonheme iron‐containing dioxygenases that respond to biotic and abiotic stress by oxidizing lipids. SiLOX7, one of the 12 LOXs found in foxtail millet, could be responsible for abiotic stress responses (Zhang et al. 2021). In addition to that, Feng et al. (2015) reported six Abscisic acid stress ripening (ASR) genes in foxtail millet, whose transcript expression patterns suggested that these ASRs may be significant in providing stress‐related signals and responding to abiotic stress in several tissues of foxtail millet. Nadeem et al. (2018) found that the increased expression of SiNRT1.1, SiNRT2.1, and SiNAR2.1, along with root architectural modifications, enhances nitrogen uptake in foxtail millet. Similarly, overexpression of the SiLEA14 gene improves salt and drought tolerance in foxtail millet, showing its potential for enhancing crop stress resistance through genetic engineering (Wang et al. 2014). Thus, it can be elucidated from these findings that millet species can tolerate harsh environments entailing abiotic stresses by adopting a combination of various genetic and enzymatic responses at the molecular level.

Millet crops are known to be robust and display resilience against various agro‐climatic challenges, including poor soil fertility and limited rainfall. Due to their exceptional adaptability, especially when compared to crops like rice, they are vital for sustaining marginal farming, a prevalent practice in hills and semi‐arid regions of India. In the regions where they are grown, these millets serve as significant sources of food grains, and their straw holds considerable value as fodder (Mal et al. 2010). In addition to that, farmers prefer to grow finger millet and sorghum because of their resistance to drought and their capability to yield with minimal resource inputs such as water and fertilizers (Yvonne et al. 2016). Thus, there is a need to highlight these traditional crops that inherently thrive in harsh arid and semi‐arid environments as a viable strategy to address climate change (Yvonne et al. 2016).

### 4.2 Millets Against Biotic Stress: Insect‐Pests and Disease

The global security of food is also at risk due to the rise and dissemination of crop pests and diseases. Human transportation plays a key role in their spread and introduction through global trade in seeds and farm products (Bebber et al. 2013). Over 90% of respondents identified an increase in diseases as one of the effects of climate change in eastern Kenya, which might have been due to enhanced habitat conditions for the infestation of pests (Yvonne et al. 2016). A growing concern that climate change is one of the major factors facilitating the establishment of pests and pathogens in newly introduced areas is becoming apparent with researchers explaining that in a warming world, crop pests and pathogens shift toward higher latitudes in both northern and southern hemispheres, with an average poleward shift of 2.7 ± 0.8 km year^−1^ since 1960 with some differences existing in pattern within taxonomic categories of insect‐pests (Bebber et al. 2013).

Several millets and their adaptation to environmental stress‐biotic and abiotic are indicated in Table 4. In Pearl millet, resistance to various pathotypes of blast pathogen *Magnaporthe grisea* has been identified, where 32 accessions demonstrated resistance (with a score of ≤ 3.0) against at least one pathotype (Sharma et al. 2013). Pearl millet seeds were also found to contain a cysteine protease inhibitor that effectively inhibits a dead wood fungus named *Trichoderma reesei*, the minimal inhibitory dose to stop mycelial development or spore germination being 1 μg mL^−1^. The antifungal activity is also demonstrated against various significant plant pathogenic fungi, including *Alternaria, Claviceps, Fusarium, Curvularia, and Helminthosporium* species (Joshi et al. 1998). The resistance of finger millet and little millet toward various insect pests can also be explained by finger millet's detrimental action on the digestive system of such insect pests. In a research carried out by Sivakumar et al. (2006), the extraction of protein inhibitors from *Panicum sumatrense* (LMCO3) and finger millet (FMCO11 and FMCO13) revealed that they were found to inhibit the digestive enzyme α‐amylases (responsible for the digestion of carbohydrates) in insect pests like *Acaea janata, Sitophilus oryzae*, and *Tribolium castaneum* , with the greatest inhibition percent recorded to be 70% for LMCO3 and 50% for FMCO13 against *Callosobruchus chinensis* . A conclusion can be derived from these findings that enzymatic responses triggered due to the prevalence of biotic stresses have been instrumental in shaping the adaptation of millet species amid the proliferation of various insect pests and diseases.

**TABLE 4:** Various millets showcasing their adaptation to biotic and abiotic stress.

Millet species	Experimental type	Experimental condition	Pigment/Gene/Enzyme	Key role for stress adaptation	References
Pearl millet	Molecular	Salt stress (NaCl; 250 mM), Oxidative stress (MV; 10 lM), cold stress (4°C), and heat stress (48°C)	Glutathione reductase (GR)	Reduces the reactive oxygen species (ROS) and regulates the homeostatic redox balance	Achary et al. (2015)
Finger millet	Molecular	250 mM NaCl, 10% PEG6000, 400 mM mannitol, water withdrawal, and heat stress	Transcription factor EcbZIP17	Tolerance to heat, drought, ABA, H_2_O_2_ stresses	Ramakrishna et al. (2018)
Foxtail millet	Genomic	Phosphorous stress, nitrate stress, drought stress, salt stress, saline‐alkaline stress, and *S. graminicola* infection	Histone acetyltransferase (HAT)	Protection against viral infection and abiotic stresses	Xing et al. (2022)
Foxtail millet	Genomic	Salt (500 mmol/L NaCl) and drought stress (< 15% soil moisture)	Plant lipoxygenases (LOXs)	Protection to stresses (biotic and abiotic)	Zhang et al. (2021)
Foxtail millet	Molecular	Three‐week‐old seedlings were immersed in 10% PEG6000, 150 mM NaCl, 200 mM mannitol, 100 lM ABA, 10 mM H_2_O_2_, and 10% sugar solutions for 3 h	Abscisic acid stress ripening (ASR) genes	Stress‐related signaling and responses to abiotic stress and oxidative stress	Feng et al. (2015)
Pearl millet	Biochemical	Protein solution from pearl millet was tested on fungal strains for fungal growth inhibition assays	Cysteine protease inhibitor	Inhibition of phytopathogenic fungi such as *Helminthosporium, Trichoderma, Alternaria*, *Claviceps, Fusarium, and Curvularia* species	Joshi et al. (1998)
Finger millet, Little millet	Biochemical	α‐amylase inhibitors from millet tested on insect‐pest α‐amylases	α‐amylase inhibitor	Reduce digestive amylolytic activities of insect‐pests like *Tribolium castaneum, Sitophilus oryzae, and Acaea janata*	Sivakumar et al. (2006)
Finger millet	Molecular	2‐week‐old seedlings were exposed to various abiotic stresses, including osmotic stress (10% PEG6000), salinity stress (80 mM NaCl), and ABA presence (100 μM ABA)	*Mannitol‐1‐phosphate dehydrogenase* (Mt1D) gene	The overexpression of mt1D in finger millet enhanced osmotic adjustment and improved chlorophyll retention under drought conditions.	Xu et al. (2020)

### 4.3 Millets and Their Assurance to Smallholder Farmers Amid Climate Change Scenarios

Millets have been a staple food and a means of livelihood in Nepal's Himalayan region for generations (Kumar et al. 2018). These resilient cereal crops thrive in challenging conditions, including the harsh climate of Nepal's high Himalayas (Sreekala et al. 2023). Millets play a vital role in reducing households' vulnerability to climate‐related risks by diversifying production and serving as a contingency crop when planting is delayed by late rains (Fischer et al. 2016). Apart from adjusting the timing of crop sowing and harvesting to cope with climatic stressors, a significant proportion (69%–73%) of subsistence farmers primarily transitioned to cultivating millets as an adaptive strategy to climate change in the Uttarakhand state of the western Indian Himalayas. This shift is attributed to the stress tolerance of millets and their importance in ensuring household food security (Shukla et al. 2019).

As a risk minimization strategy amid climate change, millets can be regarded as low‐input alternatives for cultivation on marginal lands (Fischer et al. 2016). For example, in pearl millet, the yield design of landraces focuses on their capacity to survive in water‐deficient (arid) regions by reducing the risk of crop failure, while improved modern varieties are bred to optimize yield potential in favorable conditions. Although high‐tillering landraces of pearl millet incur a production penalty under optimal conditions compared to modern low‐tillering cultivars, they are better equipped to mitigate crop failure, particularly when drought stress occurs mid‐season. For subsistence farmers in arid regions, where ideal growing conditions are rarely encountered, the adaptive techniques of landraces offer a practical solution, despite their reduced yield potential in optimal environments (Oosterom et al. 2003). Moreover, millet species contribute significantly to local food culture as they are utilized to make various cultural cuisines and beverages (Mal et al. 2010). In eastern Kenya, extension service providers have been actively directing farmers' focus toward climate change, its impacts, and strategies for both adaptation and coping (Yvonne et al. 2016). While some farmers grow these crops, they often overlook them as adaptation measures to climate change and instead limit them to subsistence production (Yvonne et al. 2016).

## 5 Way Forward and Recommendations

The conservation of germplasm resources is crucial for preserving genetic diversity within a species (Salgotra and Chauhan 2023). This diversity provides a critical foundation for breeding improved crop varieties with desirable traits, including higher yields, greater disease resistance, and enhanced nutritional quality (Swarup et al. 2021). The International Crops Research Institute for Semi‐arid Tropics (ICRISAT) gene bank serves as a global depository for millets, comprising 24.7% of total accessions belonging to millets (Table 5), which represent a key strength for advancing millet improvement programs. ICRISAT conserves around 10,193 germplasm accessions of all small millets from 50 countries (Goron and Raizada 2015). For effective utilization in crop improvement programs, core and mini‐core collections have been developed (Upadhyaya et al. 2014), which enable efficient and strategic use of the extensive native genetic diversity preserved in germplasm resources. These collections could be usefully assessed to identify sources of germplasm traits for their enhanced application in breeding highly productive cultivars with diverse adaptations (Upadhyaya and Vetriventhan 2018). Despite this structured approach, the full potential of these collections remains largely untapped, with many valuable alleles and traits still awaiting discovery, characterization, and integration into modern breeding programs.

**TABLE 5:** Germplasm subsets of millets that are developed at ICRISAT.

Crop	Germplasm subsets collection	Accessions used (no.)	Traits (no.)	References
Finger millet	Core collection	5940	14	Upadhyaya (2008)
	Mini‐core collection	622	20	Upadhyaya et al. (2010)
Foxtail millet	Core collection	1474	23	Upadhyaya et al. (2006)
	Mini‐core collection	155	21	Upadhyaya, Ravishankar, et al. (2011)
Pearl millet	Core collection	2094	22	Upadhyaya et al. (2009)
	Mini‐core collection	238	18	Upadhyaya et al. (2009)
Sorghum	Core collection	2247	21	Upadhyaya et al. (2009)
	Mini‐core collection	242	21	Upadhyaya et al. (2016)
Proso millet	Core collection	833	20	Upadhyaya, Sharma, et al. (2011)
Barnyard millet	Core collection	736	21	Upadhyaya et al. (2014)
Kodo millet	Core collection	656	20	Upadhyaya et al. (2014)

Recent advancements in genomic research have opened new avenues for enhancing crop resilience and productivity in millets. Sequenced genomes for finger millet, pearl millet, foxtail millet, and sorghum have laid a strong foundation for targeted crop improvement (Kumar et al. 2021). For instance, a genome‐wide association study identified markers linked to blast disease resistance in foxtail millet (Li et al. 2021), while QTLs associated with blast resistance in finger millet were identified through association mapping (Ramakrishnan et al. 2016). Similarly, different molecular markers have been created and deployed for QTLs/genes identification and marker‐assisted breeding to improve the breeding of pearl millet (Satyavathi et al. 2021). Such progress advances molecular breeding efforts, accelerating the development of resilient and high‐performing millet varieties. Similarly, next‐generation sequencing (NGS) technologies have enabled the development of both simple sequence repeat (SSR) and single‐nucleotide polymorphism (SNP) markers. For instance, Gimode et al. (2016) utilized NGS in finger millet to identify 80 polymorphic SNPs across 30 wild germplasm and 59 cultivated accessions, revealing significant genetic diversity that can be harnessed for breeding programs (Table 6). Around 400,000 SNP markers and 35,000 simple‐sequence repeats (SSRs) were identified from proso millets (Yue et al. 2016). Similarly, Ramesh et al. (2024) integrated gene‐based markers into a pearl millet genetic map, identifying candidate genes underlying drought tolerance QTLs. A genetic linkage map of foxtail millet using 213 SSR and InDel markers revealed 46 QTLs for key agronomic traits, with candidate genes such as Seita.9G020100 (CCT motif, heading date) and Seita.5G404900 (GA20 oxidase, plant height) identified for marker‐assisted improvement (Gao et al. 2025). These advances demonstrate the potential of molecular markers to expedite the breeding of millets.

**TABLE 6:** Molecular markers for genotyping applications in millets.

Millet	Markers	Method	Major findings	References
Finger Millet	49 SSR, 92 SNP	GBS with Roche 454 and Illumina platforms	Developed 10,327 SSRs and 23,285 SNPs; 49 SSRs and 80 SNPs were polymorphic with mean PIC 0.42 and 0.29, respectively	Gimode et al. (2016)
	9 RAPD, 5 SSR	PCR‐based marker assay	Average of 6.33 bands per RAPD primer and 7.8 bands per SSR primer; markers were able to distinguish among 40 landraces, indicating moderate genetic diversity	Joshi et al. (2020)
	15 ISSR	PCR‐based marker assay	96 scorable bands with 91.67% polymorphism with a mean PIC value of 0.307	Venkatesan et al. (2021)
Little Millet	50 tested genic SSR	Transcriptome sequencing using Ion Torrent S5 platform	24 polymorphic loci with high cross‐species transferability and an average PIC of 0.57	Desai et al. (2021)
Foxtail Millet	733 genomic SSR	Genome‐wide microsatellite variant analysis	Identified 733 highly polymorphic SSR loci; demonstrated high cross‐genera transferability (89%) to other millet and non‐millet species	Zhang, Tang, et al. (2014)
	100 genomic SSR	Genome‐wide sequencing	SSRs mapped across 1654 cM with 16.4 cM average spacing	Jia et al. (2009)
Pearl Millet	8 SSCP‐SNP	SNP detection using Illumina sequencing and SSCP profiling	Novel SNPs with mean PIC 0.49; strong synteny with related cereals	Bertin et al. (2005)
Kodo Millet	3461 SNP	GBS with Illumina platform	7 putative subpopulations of kodo millet identified	Johnson et al. (2019)
Proso Millet	1882 SNP	GBS with Illumina platform	8 putative subpopulations of kodo millet identified	Johnson et al. (2019)
Barnyard Millet	51 SSR	Transcriptome sequencing with Illumina NextSeq 500 platform	4159 protein‐coding and 2258 lncRNA transcripts were identified that are linked to drought tolerance and nutrient (Fe and Zn) accumulation	Jayakodi et al. (2019)

Recent advances in genome editing have enabled the successful implementation of zinc finger nucleases (ZFNs), transcription activator‐like effector nucleases (TALENs), and CRISPR/Cas9 systems to develop new and improved crop varieties. However, compared to mainstream cereals, millets are far less studied in terms of functional genomics (Ceasar 2022). Among different genome editing tools, the CRISPR/Cas9 system has been extensively utilized for precise genome editing because it's simple, user‐friendly, and cost‐effective construct design (Zhang et al. 2018; Van Eck 2020; Ceasar 2022). Lin et al. (2018) conducted the first genome‐editing study in foxtail millet using CRISPR/Cas9, targeting the *phytoene desaturase* (PDS) gene with the plasmid *pCAMBIA1300‐35s‐Cas9‐OsU3‐SiPDS*. CRISPR‐Cas9 technology has been successfully used in foxtail millet to edit the SiMTL gene, resulting in the development of a haploid inducer line (Cheng et al. 2021). Similarly, CRISPR/Cas9 has been successfully used to target genes like SiFMBP, SiDof4, SiBADH2, SiGBSS1, and SiIPK1 in foxtail millet, achieving up to 100% mutagenesis in the T_0_ generation (Liang et al. 2022). In broomcorn millet, a CRISPR/Cas9‐mediated system was developed to produce herbicide‐resistant plants by targeting the PmPDS gene (Liu et al. 2024). Furthermore, base editing systems (cytosine and adenine base editors) have also been utilized in foxtail millets to create nicosulfuron herbicide‐tolerant mutants by targeting genes such as SiALS and SiACC (Liang et al. 2022).

Strengthening millet cultivation through research, innovation, and policy reform is vital for building resilient food systems under climate change conditions. The application of biodiversity conservation strategies, land restoration programs (Muluneh 2021), along with organized sensitization and education campaigns, might be a good starting point for the commercialization of millets (Chhogyel and Kumar 2018). In India, Farmer‐Producer Organizations (FPOs) have played a significant role in enhancing the millet value chain by facilitating processing, collective marketing, establishing local processing units, and promoting value‐added products (Khushwaha et al. 2023). This collective approach has enabled farmers to access better markets and receive higher prices for their produce (Rafi and Tengli 2023). Similarly, participatory and asset‐based millet innovation models offer a scalable pathway to strengthen farmer resilience and livelihoods under increasing climate variability. A field trial in India showed that combining new high‐yielding millet varieties with mechanization, intercropping, and village‐level processing significantly raised the human livelihood index from 1.80 to 3.97 (Chapke et al. 2026). The pangenome concept provides a powerful framework for comprehending the full genetic composition of a species; however, its application to small millets remains largely unexplored (Kumari et al. 2024). This strategy has proven effective in other crops and could substantially speed up the genetic enhancement of small millets. To ensure the effective utilization of core and mini‐core collections in millets (Table 5), it is essential to integrate high‐throughput genotyping, precise phenotyping, and genomics‐assisted breeding approaches. Systematic characterization of these collections using molecular markers, genome‐wide association studies (GWAS), and pan‐genome analysis can help identify valuable alleles associated with key traits such as stress tolerance, yield stability, and enhanced nutritional quality, which can be efficiently incorporated into modern breeding programs. Bridging the information gap in climate science through targeted education and capacity‐building initiatives is critical for empowering policymakers, farmers, and extension workers to implement effective adaptation strategies. Collaborative efforts between the public and private sectors are also necessary to enhance knowledge dissemination and support climate‐resilient agricultural practices. Additionally, policy interventions must simplify bureaucratic processes related to seed registration and germplasm exchange in alignment with ITPGRFA guidelines, while addressing market constraints through mechanisms such as minimum support pricing (MSP) and improved commercialization strategies for neglected and underutilized species (NUS). As highlighted by the International Year of Millets 2023, fostering millet cultivation through education, land restoration, and strategic marketing is crucial for transitioning toward more resilient and diversified food systems, securing livelihoods, and climate resilience for vulnerable populations (FAO 2023). Consequently, by unlocking the full potential of these miracle grains through research and development, we can strengthen food security and enhance global well‐being under climate change.

## 6 Conclusion

Millets offer a vital, climate‐resilient solution to the growing threats of food insecurity under climate change. Their inherent resilience enables millets to thrive on marginal lands with minimal inputs, making them sustainable crops for ensuring stable yields under harsh environmental conditions. Rich in dietary fiber, essential amino acids, vitamins, and storage proteins, millets can help reduce malnutrition and play a functional role in combating chronic diseases, making them an ideal dietary component. Their broad climatic adaptability makes them an ideal model crop for research on biotic and abiotic stress tolerances. The expanding scope of value‐added millet‐based products further strengthens their role in diversified diets, market development, and livelihood improvement for smallholder farmers. Furthermore, various studies have been conducted to identify genomic regions conferring stress resistance to drought, heat, salinity, insect pests, and disease‐causing pathogens. At the same time, recent advances in conventional and molecular breeding approaches provide new avenues to improve yield potential, stress tolerance, and end‐use quality, thereby overcoming long‐standing production and adoption constraints. However, they remain underutilized, largely due to the dominance of resource‐intensive staple crops. Therefore, coordinated efforts in research, policy formulation, and market development are essential to integrate millets into agricultural systems and food supply chains, which can substantially enhance biodiversity, ecological sustainability, dietary diversity, and secure a more resilient and food‐secure future for generations to come. Future strategies should prioritize the genetic improvement of millet varieties through scientific breeding, the advancement of processing technologies, stronger policy support, and increased global awareness of their benefits. As climate variability and population growth continue to influence food production systems, the expanded cultivation and improvement of climate‐resilient crops, such as millets, are likely to play a crucial role in climate‐adaptive cropping strategies and diversified farming systems, while stabilizing future global food systems.

## Funding

The authors have nothing to report.

## Consent

The authors have nothing to report.

## Conflicts of Interest

The authors declare no conflicts of interest.

## Data Availability

The authors have nothing to report.
